# Common questions and misconceptions about diets: what does the scientific evidence really show?

**DOI:** 10.1080/15502783.2026.2707252

**Published:** 2026-10-15

**Authors:** Jose Antonio, Brandi Antonio, Alan Aragon, Shawn M. Arent, Bill Campbell, Darren G. Candow, Hannah Eberhardt, Guillermo Escalante, Cassandra Evans, Scott Forbes, Drew Gonzalez, Chad M. Kerksick, Erik Bustillo, Kworweinski Lafontant, Gianna Mastrofini, Scotty Mills, Michael J. Ormsbee, Kieran Paterson, Flavia Pereira, Valeria Silva Ramirez, Antonella Schwarz, K. Michelle Singleton, Abbie Smith-Ryan, Jeffrey Stout, Grant Tinsley

**Affiliations:** a Department of Health and Human Performance, Nova Southeastern University, Davie, FL, USA; b College of Health Professions and Sciences, University of Central Florida, Orlando, FL, USA; c Fit Advancement, LLC., Chatsworth, CA, USA (Aragon); d Arnold School of Public Health, University of South Carolina, Columbia, SC, USA; e Performance & Physique Enhancement Lab, Exercise Science & Kinesiology Program, University of South Florida, Tampa, FL, USA; f Faculty of Kinesiology and Health Studies, University of Regina, Regina, SK, Canada; g Institute of Sports Sciences & Medicine, Florida State University, Tallahassee, FL, USA; h College of Natural Sciences, California State University, San Bernadino, CA, USA; i Department of Physical Education Studies, Brandon University, Brandon, MB, Canada; j Department of Kinesiology, Sam Houston State University, Huntsville, TX, USA; k College of Science, Technology, and Health, Lindenwood University, St. Charles, MO, USA; l Train 8Nine., Miami, FL, USA; m University of Regina, Regina, SK, Canada; n Department of Health Sciences, Rocky Mountain University of Health Professions, Provo, UT, USA; o Department of Sport and Exercise Sciences, Barry University, Miami Shores, FL, USA; p Department of Kinesiology, Coastal Carolina University, Conway, SC, USA; q Department of Exercise and Sport Science, University of North Carolina, Chapel Hill, NC, USA; r Department of Kinesiology & Sport Management, Texas Tech University, Lubbock, TX, USA

**Keywords:** Calories, macronutrients, protein, nutrition

## Abstract

The study of diet and nutrition is often filled with numerous myths and misconceptions. A Google Scholar search of “diets health” yields over 4 million results. This review examines frequently asked questions about diets and dietary habits. These questions include the following: 1) Is a caloric surplus necessary for building skeletal muscle? 2) Is it possible to lose body mass or fat mass without caloric restriction? 3) Is intermittent fasting better than chronic caloric restriction for weight loss? 4) Are there sex differences in the response to fasting? 5) Can diet alone fulfill the macro and micronutrient needs of exercise-trained individuals? 6) Is there an ideal dietary macronutrient ratio? 7) Are most of the benefits of diets in overweight/obese individuals due primarily to the weight loss itself? 8) Do DNA-based diets induce greater fat mass loss? 9) Does eating late at night result in a gain in fat mass? 10) Are there data (i.e. observational, randomized controlled trials, etc.) on the carnivore diet? 11) Is there a diet that is best for healthy aging and longevity? 12) Is there such a thing as “superfoods?” 13) Can one consume adequate protein on a vegan diet? 14) Do high-protein diets harm the kidneys? 15) Does a greater meal frequency lead to greater weight loss? 16) Are carbohydrates more “fattening” than dietary fats? To answer these questions, we conducted an evidence-based scientific evaluation of the literature on these topics regarding the diet category.

## 1. Introduction

The size of the “diet” industry depends on how it is defined. For instance, the global weight management market is estimated to be a $428 billion dollar industry in 2025 [1]. Subcategories of the diet industry, such as “functional foods” and “protein,” are estimated to be valued at $40–50 billion dollars [2]. With such a large industry, it is not surprising that common misconceptions about diet remain pervasive. These range from fundamental issues, such as the validity of caloric restriction or surplus as it relates to body weight loss or gain (i.e. the energy balance model), to the antiquated notion that high-protein diets are harmful to kidney function. Debates over the superiority of intermittent fasting versus chronic caloric restriction, the existence of an “ideal” macronutrient ratio, and the legitimacy of DNA-based or plant-exclusive diets are also often questioned. Moreover, it is necessary to separate data acquired from the study of exercise-trained individuals versus those who are sedentary. Therefore, this review aims to provide an evidence-based assessment of common misconceptions about diets. These questions include the following: 1) Is a caloric surplus necessary for building skeletal muscle? 2) Is it possible to lose body mass without caloric restriction? 3) Is intermittent fasting better than chronic caloric restriction for weight loss? 4) Are there sex differences in the response to fasting? 5) Can diet alone fulfill the macro- and micronutrient needs of exercise-trained individuals? 6) Is there an ideal dietary macronutrient ratio? 7) Are most of the benefits of diets in overweight or obese individuals due primarily to the weight loss itself? 8) Do DNA-based diets induce greater fat mass loss? 9) Does eating late at night result in a gain in fat mass? 10) Are there data (i.e. observational, randomized controlled trials, etc.) on the carnivore diet? 11) Is there a diet that is best for healthy aging and longevity? 12) Is there such a thing as “superfoods?” 13) Can one consume adequate protein on a vegan diet? 14) Do high-protein diets harm the kidneys? 15) Does a greater meal frequency lead to greater weight loss? 16) Are carbohydrates more “fattening” than dietary fats? To answer these questions, we conducted an evidence-based scientific evaluation of the literature on these topics vis-à-vis diets. It should be noted that this manuscript is part of a series of articles that have sought to clarify controversial issues in the field of exercise science and sports nutrition [3–7].

### 1.1. Is a caloric surplus necessary for building skeletal muscle?

In the literature, *maintenance* has typically been discussed in the context of maintaining weight loss. For example, Stevens et al. [8] defined maintenance as a weight change of less than ±3% of the designated body weight under standardized conditions. In the present article, the definition of maintenance is more specific to body composition. Therefore, maintenance would refer to the relative stasis of lean mass and fat mass. Timeframes of 1–2 years are most commonly suggested for characterizing a state of bodyweight maintenance [9]. For the purposes of our discussion, it is reasonable to posit that a given body composition held constant for at least 6 months (a common minimum weight stability prerequisite for participants in diet research) sufficiently represents maintenance. Shorter timeframes would be more susceptible to trajectories of change in one direction or another. Garthe et al. [10] examined different nutritional approaches in elite athletes within a mix of sports (rowing, kayaking, soccer, volleyball, taekwondo, skating, and ice hockey). This study was done during the off-season, with trial periods of 8–12 weeks. The effect of nutritional counseling (resulting in a 544-kcal increase from baseline intakes) was compared with *ad libitum* dietary consumption (resulting in a 124-kcal decrease). Although the nutritional counseling group had a slightly greater increase in lean mass compared with control (1.7 versus 1.2 kg), there was also significantly greater fat gain (1.1 versus 0.2 kg). The main caveat of this trial is that the resistance training protocol was assigned in addition to the athletes' customary endurance and technical skill regimens. This may have resulted in the *interference effect* (a mitigation of resistance training adaptations, including muscle hypertrophy) [11]. More recently, Helms et al. [12] conducted an 8-week comparison of a 0%, 5%, and 15% caloric surplus in well-trained subjects. Strength gains within and between groups were insignificant except for the 15% surplus group, which experienced significant improvement in 1RM strength in the bench press. Both surplus groups increased skinfold thickness, a proxy for subcutaneous fat mass, with a slightly greater increase in the 15% versus the 5% surplus group. Biceps thickness increased slightly (~0.2–0.3 cm) versus baseline, with no significant growth differences between groups. These disappointing results have important caveats to consider. Firstly, attrition was high due to COVID-19 lockdowns. Secondly, the training protocol was significantly decreased from the subjects' habitual training volume. Finally, the reported intakes of each group were substantially greater than the assigned intakes. The groups assigned to a 0% surplus consumed an average of an additional 169 kcal, which was 7.2% beyond maintenance needs. The 5% surplus group consumed an average of 489 kcal (18.5%) beyond maintenance needs. The 15% surplus group consumed an average of 719 kcal (28.4%) beyond maintenance needs. In sum, a substantial drop in training volume combined with robust caloric surpluses unsurprisingly resulted in mostly fat gain.

The fear of undue fat gain via purposeful hypercaloric conditions is challenged by Rozenek et al. [13], who reported exclusively lean mass gains in 8 weeks despite massive caloric surpluses (~2000 kcal beyond baseline intakes). Despite the possibility that novices placed on a progressive resistance training program can experience lean gains on massive surpluses, the results of this study have not been replicated, so caution is warranted when extrapolating the findings. In contrast, Ribeiro et al. [14] compared the 4-week effects of moderate (4501 kcal) versus high-energy (6087 kcal) diets in elite male bodybuilders. The high-calorie diet resulted in greater lean mass gains (1.0 versus 0.4 kg), but also greater fat mass gains (7.4% versus 0.8%). Thus, it appears that training status may impact energy partitioning.

The composition of additional calories beyond maintenance can determine the nature of the body composition changes. This is especially true for protein. Furthermore, the outcomes can differ drastically, depending on training status and trial conditions. Bray et al. [15] reported that non-training subjects confined to a metabolic ward setting simultaneously gained lean mass and fat mass alongside escalating protein (and total energy) intakes. In contrast, a succession of studies by Antonio, et al. [16–19] showed that in free-living conditions, resistance-trained subjects do not gain body fat (i.e. −0.2 kg in one trial [20] and −1.6 kg in another trial [21] despite the addition of ~400–800 kcal of protein to baseline maintenance intakes, resulting in daily protein intakes ranging 3.3–4.4 g/kg of bodyweight per day. One of these trials [17] reported lean mass gain and significantly greater fat mass reduction in the high-protein (3.4 g/kg) versus the normal protein group (2.3 g/kg). It is possible that this phenomenon is due to increased satiety leading to spontaneous decreases in the consumption of other macronutrients, and/or increases in exercise energy expenditure through both exercise and non-exercise pathways. Regardless of what might underpin the findings, the consistency of “surplus” protein intake's ability to mitigate fat gain in these conditions is remarkable. The manufacturing and maintenance of new skeletal muscle tissue is metabolically expensive. This includes meeting the energy costs of increased muscle protein turnover, escalating training volumes, and overcoming compensatory increases in non-exercise activity thermogenesis (NEAT) [22,23]. The closer an individual is to reaching their maximum potential for muscle growth and/or leanness, the more carefully one should plan a calorie surplus to optimize muscle gain. It is noteworthy that novice and intermediate-level trainees can experience *recomposition* (simultaneous increase in lean mass and decrease in fat mass) in the absence of a purposefully targeted caloric surplus [24]. Beginning trainees have the additional advantage of being more resistant to fat gain with aggressive caloric surpluses. However, these favorable changes diminish as the individual's training status advances. From a practical perspective, it is fair to speculate that men whose body fat percentage is at or below the mid-teens (or women at or below the mid-20s) are most likely to benefit from implementing a caloric surplus to the order of ~10%–20% above maintenance requirements. This translates to ~250–500 kcal, but may fall outside of this range, depending on the individual's size and energy demands. Those prone to a high NEAT response to increased intake may occasionally require surpluses beyond this range. For those seeking to minimize fat gain amidst hypercaloric conditions, erring on the side of caution would involve staying closer to a 10% surplus, or predominating the additional calories with protein.

*In summary, hypercaloric strategies can promote skeletal muscle gain. Nonetheless, outcomes vary with training status, surplus size, and macronutrient composition. It should be noted that novice and intermediate-level trainees can improve body composition (i.e., simultaneously increase lean mass with a decrease in fat mass). Practically, a 10–20% caloric surplus, emphasizing protein, helps minimize fat gain, especially for lean or advanced trainees, and is a viable strategy for skeletal muscle accretion.*

### 1.2. Is it possible to lose body mass or fat mass without caloric restriction?

It is well known that maximizing weight loss generally requires a calorie deficit, since body weight and body composition are primarily determined by the balance between energy intake and energy expenditure. This fundamental concept, known as the energy balance model, is based on the First Law of thermodynamics, which states that energy cannot be created or destroyed, but only converted or stored [25]. If caloric intake equals caloric expenditure, body weight and composition typically remain relatively stable, indicating an energy balance. Conversely, an excess or deficit of calories, respectively, leads to weight gain or loss in the form of fat, muscle, glycogen, or water [25].

However, research indicates that significant body weight reduction can also occur without explicit caloric restriction by manipulating dietary macronutrient distribution, particularly by changing protein and carbohydrate intake. Pescari et al. [26] conducted a 12-week clinical trial involving obese women who consumed diets without intentional caloric restriction but varied in macronutrient composition. Participants were randomized into groups including a low-carbohydrate diet (LCD; ~11%–25% energy from carbohydrates, 15%–25% protein, remaining calories from fats), a ketogenic diet (KD; ≤ 10% energy from carbohydrates and maximum 50 g/day, 60% fat, 30% protein), and a high-protein diet (HPD; 40% protein, 30% carbohydrates, 30% fats), and compared against groups following calorically restricted diets. Remarkably, the LCD, KD, and HPD groups achieved greater weight loss (−3.01, −2.38, and −2.88 kg, respectively) than those on energy-restricted diets, demonstrating the efficacy of macronutrient adjustments independent of caloric reductions [26].

High-protein diets (HPD) have strong evidence supporting their effectiveness in promoting weight and fat loss, even under ad libitum eating conditions. Wycherley et al. [27] performed a meta-analysis of 24 randomized controlled trials (RCTs), totaling 1063 participants, comparing HPDs (1.07–1.60 g protein/kg/day) to standard-protein diets (SPDs; 0.55–0.88 g protein/kg/day). Despite minimal caloric intake differences (<298 kcal/day), individuals on HPDs experienced significantly greater reductions in body weight (−0.79 kg), fat mass (−0.87 kg), and some gains in fat-free mass (+0.43 kg); this was attributed to protein's higher thermic effect and greater satiation [28].

Similarly, lower-carbohydrate diets have consistently demonstrated efficacy for weight loss, independent of caloric restriction. Skov et al. [29] reported significantly greater weight loss in obese individuals following a high-protein, low-carbohydrate diet ad libitum (8.9 kg weight loss) compared to a high-carbohydrate group (5.1 kg weight loss), likely due to enhanced satiety and decreased overall energy consumption despite unrestricted intake.

Interestingly, high-carbohydrate diets can also facilitate weight loss when consumed ad libitum under certain conditions. Saris et al. [30] examined individuals consuming a low-fat, high-carbohydrate diet without caloric limitations, reporting significant reductions in body weight and fat mass. Specifically, an ad libitum diet low in fat and high in complex carbohydrates reduced body weight by 2.6 kg and fat mass by 2.4 kg compared with a moderate-fat control diet. A diet low in fat but higher in simple carbohydrates also significantly reduced body weight and fat mass compared with a control diet (1.7 and 1.9 kg, respectively). The proposed mechanism involves reduced dietary fat intake, promoting energy deficits through decreased caloric density and enhanced satiety driven by fiber-rich carbohydrate sources [30].

The potential of dietary macronutrient adjustments to drive body weight reductions without caloric restriction is supported by hormonal and metabolic mechanisms. For example, higher protein intake elevates satiety hormones such as glucagon-like peptide-1 (GLP-1) and peptide YY (PYY), reduces ghrelin secretion, and increases diet-induced thermogenesis, thereby lowering overall energy consumption and increasing energy expenditure [31]. Additionally, Rolls et al. found that high-protein and high-starch foods produced greater satiety than high-fat, high-sucrose, or mixed-content foods [32].

Moreover, evidence suggests that macronutrient composition influences the body's adaptive thermogenic response, suggesting metabolic advantages that may sustain weight loss independent of caloric reduction [25,33]. Although altering macronutrient ratios can effectively induce weight loss without direct caloric restriction, individual responses vary widely, and dietary adherence can significantly impact long-term outcomes. Prolonged low-carbohydrate intakes, for example, might pose challenges to adherence due to restrictive dietary options [34].

*In summary, although creating a caloric deficit remains ideal for optimal weight management, substantial evidence indicates that modifying the distribution of dietary macronutrients can independently facilitate significant body weight and fat loss. High-protein, low-carbohydrate diets have consistently demonstrated efficacy in promoting favorable changes in body composition.*

### 1.3. Is intermittent fasting better than chronic caloric restriction for weight loss?

With various intervals between fasting and feeding windows, there has been an influx of terms that appear similar to intermittent fasting (IF) but differ in implementation. A recent international group of experts convened to reach a consensus on the definitions of fasting [35]. They concluded that IF is defined as repetitive fasting periods lasting ≤48 hours and includes alternate-day fasting and time-restricted eating (Figure 1) [35]. Conversely, chronic caloric restriction (CCR), sometimes referred to as continuous energy restriction, represents an alternative approach where caloric intake is reduced continuously without necessarily fasting [35]. An important distinction between IF and CCR is the focus on caloric intake: IF is a scheduled regimen of feeding and fasting windows and does not specify any certain reduction in caloric intake [35]. Rather, the rationale behind IF is that a reduced window for eating would lead to a reduction in calories consumed. Meanwhile, CCR is typically used as a set reduction in caloric intake, either relative to a baseline level (i.e. “maintenance calories”) or an absolute number of calories (e.g. consuming 250 kcal less per day), taking into consideration an individual's age, sex, body composition, goals, and physical activity level [35].

**Figure 1.**
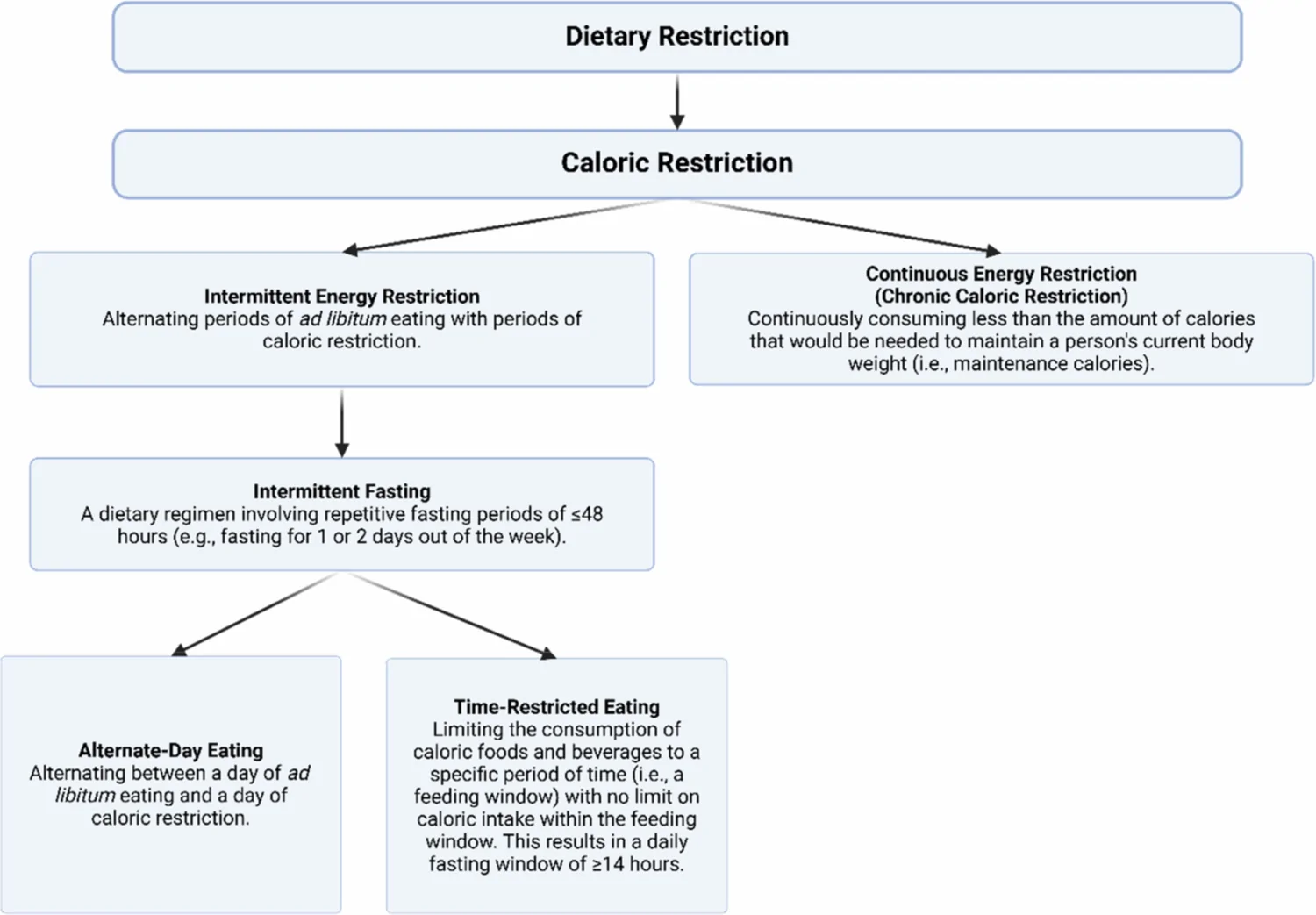
Definitions of fasting terminology according to international consensus [35,36].

Whether IF is more effective than CCR for weight loss has sparked considerable interest, both within the scientific community and among individuals seeking effective dietary strategies. While CCR has been the traditional model for weight-loss interventions, IF has been promoted as an alternative for inducing weight loss while positively affecting fat-free mass (FFM) retention, fat metabolism, and diet adherence. However, there is contention that IF is neither more nor less effective than CCR [37]. While both methods are used to reduce calorie intake and aid in weight loss, they may have distinct advantages and drawbacks.

Studies consistently show that both IF and CCR effectively promote weight loss. A systematic review and meta-analysis of randomized controlled trials (RCTs) found that IF led to slightly greater short-term weight loss than CCR, with an average additional reduction of about 0.94 kg over 6 months [38]. While this difference is statistically significant, it is modest and may not have much clinical relevance. However, long-term studies indicate that the gap between IF and CCR tends to narrow over time, with both methods achieving similar weight loss results when maintained for more than six months [39]. These findings are supported by another systematic review that reported comparable long-term weight-loss outcomes for both approaches, despite IF's slight edge in the short term [38]. Additionally, an umbrella review noted that IF was more effective than CCR in reducing waist circumference and fat mass, providing strong evidence for these improvements [40].

One of the proposed benefits of IF is its ability to reduce fat while preserving fat-free mass (FFM). Research suggests intermittent energy restriction (IER), including IF, may lead to better fat-to-FFM ratios than CCR. A meta-analysis found that participants following IF lost more fat while maintaining their fat-free mass more effectively than those on CCR [41]. This could be due to fasting-induced metabolic changes, such as increased fat breakdown and reduced reliance on glycogen stores. Supporting this, recent studies indicate that IF leads to a modest yet significant reduction in fat mass compared to CCR in short-term interventions [38]. Additionally, an umbrella review revealed that IF significantly increased FFM, underscoring its potential to help preserve lean body composition during weight loss efforts [40].

The benefits of IF may go beyond just weight loss; they also significantly enhance insulin sensitivity and glucose metabolism. Research shows that short-term fasting can notably lower fasting insulin levels and the homeostatic model assessment of insulin resistance (HOMA-IR) compared to CCR [38]. These improvements could offer extra health perks, especially for people with insulin resistance or metabolic syndrome. Both IF and CCR effectively reduce blood pressure, triglycerides, and cholesterol levels, indicating that they provide similar benefits in lowering cardiovascular risk [39]. It is worth noting that an umbrella review found that IF was less effective than continuous energy restriction in reducing systolic blood pressure, although it did help improve levels of HDL cholesterol, triglycerides, and total cholesterol [40].

Adhering to dietary interventions is essential for their long-term success. Intermittent fasting offers a more straightforward approach, as it generally does not require detailed calorie counting, a task that some people might find overwhelming. Instead, it focuses on eating within specific time frames or alternating between fasting and feeding days. While this simplicity can be appealing, some individuals may struggle with hunger and the mental challenges of prolonged fasting [41]. On the other hand, CCR requires consistent daily calorie reduction, which can be effective but may lead to feelings of deprivation over time, potentially making it more challenging for people to adhere to it. Long-term studies on adherence emphasize the importance of individual preferences and the need for tailored dietary strategies to improve sustainability [39].

The effectiveness of IF compared to CCR can vary based on personal factors, such as metabolic health, lifestyle, and individual preferences. For example, people with metabolic syndrome or type 2 diabetes might see more significant improvements with IF because it strongly affects insulin sensitivity [38]. On the other hand, those who prefer regular eating schedules might find CCR easier to stick with [41]. Furthermore, subgroup analyzes from an extensive umbrella review indicate that IF could be especially beneficial for individuals with obesity or metabolic syndrome, particularly in enhancing fasting insulin levels and HOMA-IR [40].

Many studies on IF and CCR are short-term, often lasting less than a year, making it hard to conclude their long-term efficacy and safety. Moreover, adherence rates and reasons for dropouts are often underreported, complicating our ability to assess how these interventions might work in real-life situations. There is a clear need for more comprehensive, long-term randomized controlled trials (RCTs) involving diverse populations to fill these gaps [39,41]. Additionally, differences in study methodologies, such as varying fasting regimens and the lack of dietary intake control in some trials, lead to inconsistencies in the findings [38]. Recent reviews highlight the necessity for large-scale, long-term studies to confirm IF's metabolic and physical benefits [40].

Both IF and CCR are effective strategies for weight loss, showing only modest differences in short-term results. IF might provide additional benefits, such as reducing fat mass and improving metabolic health, particularly in terms of insulin sensitivity. However, its sustainability largely depends on personal preferences and one's ability to handle fasting periods. On the other hand, CCR involves consistently reducing daily calorie intake, a well-established method that can be difficult to stick with long-term due to feelings of deprivation [38].

*In summary, choosing between IF and CCR should be based on individual needs and preferences. A personalized approach considering psychological and lifestyle factors is crucial for ensuring long-term commitment and lasting weight loss. Future research should focus on clarifying the long-term effects of these dietary strategies, helping both individuals and healthcare providers make informed choices tailored to specific health goals* [38–41].

### 1.4. Are there sex differences in the response to fasting?

Various fasting paradigms remain popular among individuals seeking to improve their health and body composition, particularly intermittent fasting protocols such as time-restricted eating [42]. These eating schedules involve repeated periods of acute fasting, during which physiological responses occur in accordance with the duration of nutrient deprivation [43]. A matter of common debate is whether there are sex differences in the responses to fasting, both acutely and chronically, with the implied question of whether the potential suitability of intermittent fasting as an eating pattern differs dichotomously based on sex.

Several studies have investigated potential sex differences in responses to acute periods of fasting [44,45]. In one investigation, healthy adult females showed a higher rate of appearance of free fatty acids (FFA) after a ~12-hour fasting period, reflecting increased lipolysis, compared with males [44]. However, there was no sex difference in fatty acid oxidation; rather, nonoxidative disposal of FFA was elevated in females, contributing to higher FFA turnover even when scaled to total resting energy expenditure. During a 48-hour fasting period, it was demonstrated that the nonoxidative disposal of FFA and post-fasting responses to nutrient ingestion may differ by sex [45]. While mean FFA concentrations were higher in females than males during the first 24 hours of fasting, an acceleration of FFA appearance was observed in males from ~20 to 32 hours of fasting, leading to more similar FFA concentrations in the later periods of the fast. Interestingly, it was reported that the proportion of nonoxidative FFA disposal across organs may differ between sexes, with greater FFA incorporation into triglycerides occurring in the liver in males but in skeletal muscle in females. Upon subsequent refeeding after the 48-hour fast, a mixed meal of ~570–670 kcal (60% carbohydrate, 25% fat, 15% protein) resulted in higher insulin concentrations in females than in males, without differences in peak glucose concentrations, indicating poorer insulin sensitivity in females [45].

Despite these physiological findings, the relevance of sex differences during acute periods of nutrient deprivation on long-term adaptations to fasting programs is unclear. In general, clinical trials have not clearly supported differential effects of intermittent fasting programs by sex [46,47]. However, a meta-analysis has indicated potential for differential improvements in blood lipids depending on whether studies included females only, males only, or mixed-sex cohorts [46]. Additionally, one trial of 22 days of alternate-day fasting demonstrated an apparent worsening of glucose clearance in females in response to ingestion of a 500-kcal liquid meal (12 g fat, 80 g carbohydrate, 18 g protein) in the fasted state [47]. The relevance of this finding to other forms of fasting and ingestion of solid foods, as well as in relation to other outcomes beyond blood glucose, is questionable. Overall, while some individual trials have demonstrated sex differences related to fasting programs, current research does not indicate a need to specifically emphasize or deemphasize the potential suitability of these programs based on sex alone.

In the context of controlled trials of intermittent fasting in exercising populations, most studies to date have included primarily males [48–51] or females [52–54] rather than balanced groups that would allow for sex-based analysis. However, one trial of 5:2 intermittent fasting plus supervised resistance training for 12 weeks included both males and females in similar proportions and observed more weight loss in males but greater increases in fat-free mass in females [55]. Nonetheless, additional trials specifically designed to examine sex differences in chronic responses to intermittent fasting practiced in conjunction with structured exercise training are warranted before firm conclusions can be made.

*In summary, although some differences in physiological responses to fasting have been observed between sexes, current evidence does not support the need to tailor intermittent fasting protocols specifically by sex.*

### 1.5. Can diet alone fulfill the macro- and micronutrient needs of exercise-trained individuals?

The macro- and micronutrient needs of exercise-trained individuals are greater than those of the general population. Protein needs range from 1.2 to 2.2 g/kg, depending on performance goals, as opposed to the 0.8 g/kg recommended for the general population [56]. Calorie and carbohydrate intake are higher in order to support the increased physical demands. Exercise-trained individuals endure physical stress and lose more micronutrients through various pathways, such as sweat, urine, and increased metabolic activity; thus, micronutrient needs are greater, especially for iron, vitamin D, magnesium, B vitamins, sodium, and potassium [57,58]. Due to the increased physical demands that exercise places on their bodies, meeting their nutritional needs through diet alone becomes a critical question. Although exact numbers are unknown, most athletes of all levels and recreationally active individuals are thought to use dietary supplements [59]. However, some nutritionists and health professionals argue that supplements are unnecessary and, at times, potentially harmful [56,60]. From a sports performance perspective, certain nutrients are well-established as safe and beneficial for enhancing performance [56]. Moreover, some of these nutrients, such as creatine, are required in greater amounts than what the diet alone can provide. Hence, supplementation is necessary for nutritional ergogenic aids. However, not all supplements offer a direct ergogenic effect. For example, multivitamins may help correct or prevent deficiencies, whereas protein supplements may assist individuals in meeting their daily protein needs. Not meeting nutritional needs negatively impacts numerous physiological processes and indirectly affects their performance.

There are numerous reasons exercise-trained individuals should integrate supplements into their training plan. In obvious instances, such as nutrient deficiencies or malabsorption, supplementation is needed to correct/restore nutrients to optimal levels. Personal taste preferences, restrictions related to religious practices, and food intolerances/allergies limit the consumption of specific foods, resulting in suboptimal intake of macro and micronutrients. Specific diets or dietary practices influence nutrient intake. Restrictive diets, which exclude or eliminate certain foods, often lack essential nutrients [61–63]. For example, vegan or vegetarian diets are often lacking in several vitamins (e.g. B12, B3, B2, D) and minerals (e.g. calcium, iodine, potassium, selenium, and zinc) [64]. Given the numerous scenarios in which dietary restrictions, personal preferences, or nutrient deficiencies limit intake of essential macro- and micronutrients, supplementation is necessary.

It is commonly believed that supplementation is not needed for those adhering to a well-rounded, balanced diet [65]; however, research suggests otherwise. Numerous studies reveal that even well-trained individuals are often lacking in essential nutrients [57,66–68]. Hinton et al. [69] reported that a significant number of NCAA Division I collegiate athletes failed to meet the recommended intake of carbohydrates and protein. Sports with lower overall energy intake, such as wrestling, gymnastics, and bodybuilding, are susceptible to micronutrient deficiencies, especially calcium and iron [66–68]. Endurance athletes, despite having higher energy and carbohydrate needs, still struggle to meet their daily needs [66]. Moss et al. [70] reported that both male and female endurance athletes did not meet the recommended intake of energy or carbohydrates. Compared with men, women had a lower protein intake, likely related to reduced energy consumption. Numerous athletes were reported to have deficient intakes of both water and fat-soluble vitamins [70]. Wardenaar et al. [57] examined the micronutrient intake and supplementation use in Dutch athletes (*n* = 553). Vitamin D intake was below the estimated average requirement in all subjects. This was true even for those who reported using supplements. Notably, athletes who were not using supplements were at risk of inadequate intake of vitamins B1, B2, B3, A, C, and selenium. In women, iron intake was below the estimated average requirement.

*In summary, athletes often fail to meet their macro and micronutrient needs. Although a well-planned, varied, and high-energy diet can theoretically meet macro- and micronutrient needs, in practice, most exercise-trained individuals struggle to do so consistently due to high energy demands, food volume requirements, cost, time constraints, and dietary preferences/restrictions. Therefore, strategic supplementation is often necessary to ensure optimal intake.*

### 1.6. Is there an ideal dietary macronutrient ratio?

The concept of an “ideal” macronutrient ratio (i.e. an optimal percentage distribution of carbohydrates, fats, and protein) has been a longstanding fixture in public nutrition messaging and dietary fads. Popular approaches such as the “If It Fits Your Macros” (IIFYM) strategy have promoted the notion that as long as macronutrient targets are met, food quality and source are secondary considerations. However, contemporary research challenges this paradigm, highlighting the complex interplay between nutrient quality, caloric intake, and physiological goals.

A common misconception is that manipulating the macronutrient ratio (i.e. carbohydrates and fats) in a diet can independently drive weight loss or prevent weight gain. A large systematic review by Fogelhom et al. [71] evaluated prospective cohort and intervention studies and concluded that macronutrient distribution (% of energy from carbohydrates, fats, or proteins) was not predictive of long-term changes in body weight or waist circumference. Instead, dietary patterns characterized by high intake of fiber, whole grains, fruits, and dairy were associated with less weight gain, while higher consumption of meat, refined grains, and sugar-rich foods predicted greater weight gain. Similarly, de Souza et al. [72] found that caloric deficit was more influential than macronutrient composition of the diet on the degree of fat loss. Metabolizable energy, a key consideration in energy balance models, is also impacted by the fiber content of food [73] as well as the degree of processing [74,75] rather than simply composition ratios of the macronutrients. This further reinforces the idea that overall food quality and energy density, rather than macronutrient ratios per se, appear to be more influential in regulating body weight.

Gardner et al. [76] randomized participants to Atkins (low carbohydrate), Zone (40:30:30 ratio of carbohydrate, protein, fat), Lifestyle, Exercise, Attitudes, Relationships, Nutrition (LEARN: (lifestyle/exercise/attitudes/relationships/nutrition/moderate low-fat, or Ornish (very-low-fat vegetarian) diets and found significant variations in micronutrient sufficiency depending on diet composition. They concluded that focusing exclusively on macronutrient ratios can lead to neglect of micronutrient adequacy, particularly when food variety is limited. Similarly, a study by Ismaeel et al. [77] on competitive bodybuilders revealed that both macronutrient-based and strict dieters frequently fell short of meeting dietary recommendations for essential micronutrients, such as vitamin D, potassium, and fiber, despite meeting their macro targets. Similarly, evaluating dietary fat solely by total percentage can obscure critical differences in health effects between saturated and unsaturated fats. Evidence consistently supports replacing saturated fat, especially that from processed meats and tropical oils, with unsaturated fats, such as monounsaturated and polyunsaturated fatty acids, which improve lipid profiles and reduce cardiovascular risk [78,79]. These findings underscore that diet quality and composition, rather than simply macronutrient distribution, play a pivotal role in achieving optimal health outcomes.

As an additional consideration, while macronutrient ratios are typically expressed as percentages of total caloric intake, this method may obscure the absolute protein needs of individuals, particularly those who are physically active or seeking to optimize body composition. The International Society of Sports Nutrition (ISSN) recommends protein intakes ranging from 1.4 to 2.0 g/kg of body weight per day (g/kg/d) for active individuals, with intakes above 3.0 g/kg/d potentially offering advantages in fat mass reduction [80]. Critically, expressing protein intake as a fixed percentage can result in underconsumption, especially in hypocaloric diets. For example, a 70-kg individual on a 1600 kcal/day diet consuming 15% of energy from protein would only ingest 60 g of protein, far below the ISSN minimum of 98 g/day. This discrepancy highlights the importance of calculating protein in absolute terms rather than as a percentage of calories.

Another layer of complexity involves matching macronutrient intake with physiological and performance goals. Athletes and active individuals may require greater carbohydrate availability during high-intensity or high-volume training phases, and protein needs vary depending on training phase, age, and body composition objectives [81]. Post-exercise nutrient timing studies show that co-ingesting protein and carbohydrates enhances muscle glycogen replenishment and recovery [81,82], with carbohydrate-protein supplements often outperforming carbohydrate alone in promoting muscle glycogen resynthesis [83]. However, the actual ratio of these macronutrients is ultimately driven by training demands and targeted outcomes. In this case, nutrient periodization, or the deliberate adjustment of macronutrient intake to align with specific training demands and recovery windows, has stronger support than an overarching macronutrient ratio.

*In summary, the prevailing emphasis on finding an 'ideal' macronutrient ratio oversimplifies the intricacies of human nutrition. Scientific evidence supports a shift toward more nuanced individualized dietary planning that prioritizes total energy balance, adequate protein intake in grams per day, nutrient-dense food choices, and alignment with physiological goals. Rather than adhering to rigid macronutrient percentages, diets should be structured to support functional outcomes such as performance, recovery, and long-term health, with flexibility to accommodate different metabolic and lifestyle needs.*

### 1.7. Are most of the benefits of diets in the overweight/obese due mainly to weight loss?

It has long been debated whether diet composition matters for weight loss or whether it is simply a matter of “calories in, calories out” (CICO) that confers the benefit [84–87]. Many researchers widely accept the CICO paradigm [86,88]. Technically speaking, CICO is valid in terms of being a central tenet for obesity prevention and management, wherein caloric restriction is the underlying mechanism that leads to weight loss and improved health-related outcomes. However, there are arguments against the simplicity of CICO, which highlight that it fails to consider the composition of the diet (i.e. macronutrient content) when it comes to weight gain or loss. This has led to several factions of researchers questioning the validity of CICO, suggesting that other paradigms should be considered, as they may lead to better weight loss outcomes (i.e. the carbohydrate-insulin hypothesis, which posits that carbohydrate restriction results in a metabolic advantage).

In the 2017 ISSN Position Stand, Aragon and colleagues [88] concluded that several dietary interventions can effectively improve body composition, which coincides with the conclusions of Howell and Kones' 2017 paper [86] that weight loss (or gain) largely depends on total daily energy intake (or calories ingested). Furthermore, Aragon and colleagues [88] noted that no controlled isocaloric diet comparison studies reported clinically meaningful weight loss advantages following a low-carbohydrate or ketogenic diet, which combats the carbohydrate-insulin model of obesity. To date, no data suggest the superiority of one diet (i.e. low-carbohydrate/ketogenic, low-fat, etc.) over another, especially when protein content is matched between study groups [89]. Hall et al. [90] noted that the energy balance model (i.e. CICO) allows one to undertake various eating patterns and macronutrient composition while ultimately sustaining a reduction in calories, which is the main driver of weight loss. Generally, one tends to improve cardiometabolic health risk factors (i.e. glycemic measures, blood lipids, inflammation, oxidative stress, etc.) with weight loss, and it has been demonstrated that a 5% reduction in weight is considered clinically meaningful [91]. In addition, Kritchevsky and colleagues [92] found that a 15% reduction in all-cause mortality was attributed to weight loss (RR = 0.85; 95% confidence interval: 0.73–1.00). Interestingly, dietary patterns that include the consumption of various foods like vegetables, fruits, nuts, whole grains, fish, and more are associated with a decreased risk of all-cause mortality (141 studies considered), while there is insufficient evidence on the relationship between a diet's macronutrient content and all-cause mortality (28 studies considered) [93]. Based on this, it does seem reasonable to suggest that one's dietary pattern, or the composition of the food groups consumed in their diet, can support health benefits, but this is still likely due to caloric restriction [94]. Collectively, these findings and conclusions from comprehensive reviews provide the logical framework behind the notion that a diet's benefits are largely the result of weight loss rather than its composition.

It is worth noting that data suggest increasing protein intake during caloric restriction can be beneficial [95]. However, is a calorie from a carbohydrate the same as one from fat or protein, and will these have the same effect on the body after consumption? Several reports suggest that protein positively affects body composition and that, even when overconsumed, it does not appear to negatively affect fat mass or body fat percentage. For example, Antonio and colleagues [20] assessed the impact of a high-protein diet (4.4 g/kg/d) for 8 weeks among 30 healthy resistance-trained individuals. Although the high-protein group consumed an additional average of  ≈ 800 kcals/d over the 8 weeks (from protein), the participants did not demonstrate increases in fat mass, body mass, or percent body fat [20]. Another study by Antonio et al. [96] assessed the effects of a lower protein (2.3 g/kg/d) and higher protein (3.4 g protein/kg/day) diet combined with periodized heavy resistance training on body composition. The higher protein group demonstrated significantly reduced fat mass (*p* = 0.04; 1.6 vs 0.3 kg) and percentage body fat (*p* = 0.05; 2.4% vs 0.6%) than the lower protein group. Interestingly, the higher protein group consumed an additional ≈400 kcals/d during the 8-week study, and these findings are corroborated by other investigations on consuming a relatively higher protein diet [97,98]. While weight loss was not the goal of these studies, it is important to consider the impact of a higher amount of protein within one's diet, as these data demonstrate a higher protein diet is more favorable in terms of body composition (i.e. accretion and retention of lean muscle mass), and this may also provide evidence to suggest that calories from protein may be processed differently than those from carbohydrates and fat. This is important to consider in terms of further augmenting the benefits of a calorie-restricted diet approach, especially when considering that a higher protein intake during the period of calorie restriction aids in retaining lean mass [95].

*In summary, the current body of literature supports the notion that most diet benefits in overweight and obese individuals result from weight loss (from caloric restriction) and not the composition of the diet* [86,89,99,100]*. However, dietary protein should be considered to augment the benefits of a weight-loss dietary approach. While CICO may be an oversimplification and debates about the role of diet composition continue, from a thermodynamic perspective, consuming fewer calories and increasing physical activity will lead to weight loss and, consequently, an improvement in all-cause mortality, regardless of the specific composition of the diet.*

### 1.8. Do DNA-based diets induce greater fat loss?

Since the completion of the Human Genome Project in 2004, scientists have made significant progress in understanding the intricate ways in which genes influence health and bodily functions. Nutrigenomics, the scientific field focused on the interaction between nutrition and genes, plays a role in exploring how diet influences disease prevention and treatment, as well as the potential reduction of body fat.

When total calorie intake and protein intake are kept consistent, individuals tend to lose similar amounts of weight whether they follow a high-carbohydrate/low-fat diet or a low-carbohydrate/high-fat diet. When reporting group-level data, the weight loss differences between these two approaches are typically minimal or insignificant. Whereas when reporting individual weight loss, responses vary substantially within the groups, indicating that some individuals may react differently to high-carbohydrate versus high-fat diets. Consistent with this individual variability, the potential for precision nutrition based on an individual's genetic data to predict phenotypes as well as response to dietary interventions has gained attention in recent years [101].

Advocates of DNA-based diets suggest individuals metabolize carbohydrates and fats differently depending on their genetic makeup. Individuals may be carbohydrate-responsive or fat-responsive, and these traits may promote greater weight loss. In support of this contention, Höchhsmann and colleagues conducted a randomized controlled trial to test the hypothesis that participants assigned to a diet corresponding to their a priori-determined genotype (fat-responsive or carbohydrate-responsive) would lose more weight over 12 weeks than those assigned to a diet discordant with their genotype [102].

The researchers conducted genetic analyzes of 122 overweight and obese individuals to identify the presence of carbohydrate-responsive or fat-responsive genes. After identifying the fat-responsive (*n* = 85) and carbohydrate-responsive (*n* = 37) groups, further subdivisions were made into the following categories: Fat-Responders on a high-fat diet, Fat-Responders on a low-fat diet, carbohydrate responders on a high-carb diet, and carbohydrate responders on a low-carb diet. All subjects were prescribed a 750-calorie deficit for 12 weeks. The high-carbohydrate diet consisted of 65% carbohydrates and 20% fat, while the high-fat diet comprised 40% fat and 45% carbohydrates. Both diets provided 15% energy from protein.

After the 12-week deficit, the researchers reported no differences in total weight loss from the fat-responsive group (high-fat diet = −5.5 kg, low-fat diet = −5.3 kg). Similar results were observed in the carbohydrate-responsive group as there were no statistically significant differences in the amount of weight loss for the higher vs. lower carbohydrate diet (high carbohydrate diet = −5.1 kg-, low carbohydrate Diet = −4.1 kg). Similar to weight change, change in body fat did not differ between genotype-concordant and genotype-discordant diets. This 12-week randomized controlled trial found no significant difference in weight loss or body fat between individuals with carbohydrate-responsive genes and fat-responsive genes. Both groups followed a high-carbohydrate diet or a high-fat diet, with specific energy targets and the same level of energy restriction.

Other research has reported similar outcomes. Coletta et al. investigated the effects of a moderate carbohydrate or lower carbohydrate hypo-energetic diet that aligned or did not align with genotype for 24 weeks while participating in a resistance training and walking program in sedentary women with obesity [103]. While adherence to the exercise and weight-loss program promoted improvements in body composition and health outcomes, alignment of these diets to their genetic profile did not promote improvements in health or body fat outcomes. Likewise, when overweight adults were randomized to a healthy low-fat vs. a healthy low-carbohydrate diet for 12 months, there was no significant diet-genotype pattern interaction for weight loss [104].

*In summary, studies show that when calorie and protein intake are equal, people lose similar amounts of weight on both high-carb/low-fat and low-carb/high-fat diets, with group-level differences being minimal. However, individual responses to these diets can vary, leading to interest in tailoring diets based on genetic profiles. Despite this, there is no current evidence that DNA-based diets may provide additional benefits over standard dietary approaches.*

### 1.9. Does eating late at night result in a gain in fat mass?

A common misconception suggests that consuming calories late at night leads to weight gain and increased fat mass. This belief has been perpetuated from two primary themes. First, eating a majority of daily calories in the late hours, just before bed, has been shown in some studies to be associated with an increased risk of obesity and corresponding diseases [105]. We also know that a larger (>500 kcals) mixed-macronutrient meal in the late hours does not induce significant satiation and has a reduced thermic effect of food compared to the same large meal eaten earlier in the day [106]. Taken together, these circumstances could increase caloric intake, leading to fat gain. Second, there is the oversimplified notion that metabolism slows significantly during sleep, causing nutrients to be stored as fat rather than oxidized for energy [107]. However, while total energy expenditure decreases during sleep due to reduced physical activity, resting metabolic rate (RMR), which accounts for as much as 60–75% of total daily energy expenditure [108], is not affected while sleeping.

Like any misconception, it is critical to understand the nuance around this topic. For example, pre-sleep feeding is defined as food eaten more than two hours after dinner and within 30 minutes of getting into bed [109]. In addition, some people exercise or compete in the evening and must also practice or compete the next day. These individuals may be required to eat before bed to avoid any issues surrounding energy availability. With that in mind, pre-sleep feeding of a protein-dominant snack (approximately 100–300 kcals; 30–40 g protein) may, in fact, lead to favorable body composition changes, provide an opportunity to reach daily nutrient needs, and affect next morning hunger cues.

Madzima et al. explored the influence of pre-sleep nutrient intake on resting metabolic rate [110]. The effects of 140–150 kcals of whey protein (WP; 30 g), casein protein (CP, 30 g), carbohydrate (CHO, 33 g), or a non-nutritive placebo 30 minutes before sleep in active young adult males were investigated [110]. The following morning, RMR was significantly elevated following the consumption of WP (+104 kcals), CP (+98 kcals), and CHO (+65 kcals) compared to placebo. This suggests that regardless of macronutrient type, pre-sleep nutrient intake may positively influence next-morning RMR compared to not eating. However, not all agree, as Lay and colleagues reported no difference in next-morning RMR following pre-sleep feeding [111]. Nevertheless, small changes to RMR due to small, protein-dominant snacks may add up over time to help, rather than harm, body composition.

Outside of total daily energy expenditure, pre-sleep nutrient intake does not appear to impair fat metabolism as measured by in situ microdialysis. Kinsey et al. observed no differences in subcutaneous abdominal adipose tissue (SCAAT) glycerol concentrations (CP: 171.6 ± 19.1 vs placebo: 161.5 ± 18.6 μM, respectively)—a key marker of lipolysis, nor did they report any changes to fat oxidation or insulin in males with obesity following one night of pre-sleep CP (30 g) consumption compared to placebo [112]. Likewise, Allman and colleagues compared lipolysis following daytime versus pre-sleep protein (30 g) intake [113]. No differences were observed in SCAAT lipolysis indicated by next-morning interstitial glycerol concentrations (daytime: 321.0 ± 77.1; pre-sleep: 333.0 ± 68.0 μM) and fat oxidation (daytime: 5.00 ± 0.28; pre-sleep: 5.44 ± 0.27 g/min) in resistance-trained females regardless of the time-of-day protein was consumed [113]. These findings suggest that pre-sleep protein consumption does not impair lipolysis or fat metabolism and may be a valuable strategy for optimizing protein intake and overall macronutrient profile.

Furthermore, consuming a pre-sleep protein bolus may present an effective strategy to improve muscle adaptive responses to exercise by increasing lean mass and improving body composition. Snijder et al. reported significant increases in skeletal muscle mass, strength, and muscle fiber size following 12 weeks of resistance training when consuming pre-sleep nutrition (27.5 g of protein, 15 g of CHO) before bed compared to a non-nutritive placebo [114]. Importantly, no changes were reported to total fat mass over 12 weeks when consuming an additional 175 kcals each night. In addition, the pre-sleep protein was a simple and effective way to increase total daily protein intake (1.9 g/kg/d) compared to the control group (1.3 g/kg/day). Therefore, pre-sleep protein consumption, when paired with resistance exercise, provides a simple solution to increase total daily protein intake and may promote muscle accretion without concurrent increases in fat mass.

The current body of scientific literature does not support the notion that late-night eating leads to weight gain and fat accumulation when referring to protein-centric snacks. Rather, pre-sleep nutrient intake, particularly in the form of a protein-dominant snack (100–300 kcal; 30–40 g protein), may offer several benefits, including a small, but significant, increase in resting metabolic rate (RMR), enhanced muscle protein synthesis, reducing the chance for energy deficiencies, maintaining lipolysis and fat oxidation, and providing an option to eat before bed when needed. Notably, consuming a pre-sleep protein bolus has been shown to support muscle adaptation to exercise, promote lean mass gains, and improve body composition without increasing fat mass.

*In summary, these findings challenge the misconception that late-night eating will make you gain fat and suggest that a more nuanced approach to nutrient timing is important in optimizing metabolic health and body composition. Further research is needed to explore “caloric cutoffs” and macronutrient combinations before sleep, particularly in different populations and with longer-term interventions.*

### 1.10. Are there data (i.e. RCT or Cohort) on the carnivore diet? What does the science reveal?

One can broadly define the carnivore diet as the avoidance of plant foods. This distinguishes it from mere carbohydrate restriction, since even a by-the-book ketogenic diet can include an abundance of plant foods and remain within the 50 g/day carbohydrate limit. Ketogenic dieting for weight loss has had widespread fanfare for over half a century [115], and a full century if you count its use for neurological diseases [116]. However, the mainstream popularity of the *carnivore* (plant-free) variant is a relatively recent phenomenon. On this note, a recent set of case reports by Norwitz and Soto-Mota [117] found that carnivore diets (comprised mostly of meat, eggs, and animal fats) were consistently effective for improving the symptoms of subjects with ulcerative colitis and Crohn's disease. These findings lend support for the justification of the carnivore diet as a baseline for an elimination/reintroduction diet to investigate foods that might be culpable for an individual's adverse responses.

Perhaps the first appearance of a carnivore (plant-free) diet in the scientific literature was in the *Journal of Biological Chemistry* in 1930, chronicling the 12-month diet of two Arctic explorers, which consisted exclusively of meat and fat [118,119]. Intake ranged from 2000 to 3100 kcal/day (approximately 15% protein, 75%–85% fat, and 1%–2% carbohydrate). Both men were lean and in good physical shape at the start of the study and lost 2.0 and 3.6 kg by the end of the 12 months, respectively. The remarkable finding was that neither subject showed any subjective or objective signs of degradation in physical or mental health (i.e. subjects maintained body weight, stayed physically active, reported no significant mental or physical complaints, and showed no clear evidence of kidney dysfunction). Interestingly, one of the subject's mild cases of gingivitis disappeared by the end of the study. This case study of Arctic explorers is often cited as evidence for the viability of an all-meat diet. However, among various internet platforms, there is a pervasive idea that an authentic or purist carnivore diet consists solely of fatty cuts of beef. Unfortunately, consuming a single-food diet, such as steak, is inherently limited in terms of the spectrum of nutrients it provides. It is noteworthy that the health-preserving diets of the Arctic explorers included a considerable range of foods. Their diets included beef, lamb, veal, pork, chicken, and bacon. The parts consumed were muscle, fat, liver, kidney, bone marrow, and brain. Therefore, to emulate these explorers, one would have to consume a far greater variety of foods than just beef steak.

The first peer-reviewed publication to examine a large sample of subjects was a 2021 observational study by Lennerz et al. [120], who surveyed an online community of 2029 adults following a carnivore diet. They were engaged in the diet for a minimum of 6 months (median duration was 14 months, range was 9–20 months). The main findings were mostly favorable, including high satisfaction with the carnivore diet and a low prevalence of perceived nutrient deficiency. Although the carnivore diet is devoid of vitamin C, deficiency symptoms (i.e. scurvy) were not reported. It should also be noted that the majority of this community (63%) took supplements. This survey has several limitations, including reliance on self-reported data (i.e. errors of recall) and the absence of a control group. Nonetheless, 95% of the participants reported improvements in overall health.

A narrative review by O’Hearn [121] speculated that the high carnitine content of the carnivore diet may decrease dietary vitamin C requirements. Expectedly, LDL cholesterol, on average, increased from borderline high to high levels. On the more favorable side, triglycerides decreased, and HDL cholesterol increased. Of potential concern is that the coronary artery calcium (CAC) score increased from 183 to 322. For reference, a CAC score from 101 to 400 indicates moderate atherosclerotic plaque, and above 400 indicates extensive atherosclerotic plaque [122]. This data does not bode well for the protection of cardiovascular health. A relatively simple modification of a typical carnivore diet would be to decrease the proportion of land animal fat and increase the proportion of fatty fish in the diet [123].

Goedeke et al. [124] examined the nutrient composition of carnivore meal plans designed to (attempt to) meet the requirements of the average Australian male and female (with an average BMI and activity level). Total energy for the male and female plans was 2120 and 2663 kcal, respectively. Protein was set at 25%–30%, fat was 70%–75%, and carbohydrate was below 5% of total calories. Dairy and non-dairy versions of the diets were created (totaling four diets for analysis). The authors tried to keep the diets ecologically valid. Thus, the diets had a ruminant-based meat selection (with lesser quantities of pork, chicken, and fish), as commonly recommended in carnivore doctrine. All meal plans exceeded nutrient reference values (NRVs) for riboflavin, niacin, phosphorus, zinc, vitamin B6, vitamin B12, selenium, and vitamin A. However, meal plans were below the recommended dietary intakes (RDIs) for thiamin, magnesium, calcium, vitamin C, iron (meal plan one only), folate (meal plans 1 and 4 only), and iodine (meal plans 1 and 3 only). Potassium intake was below adequate intakes (AI) in meal plans 1, 2 (98% of AI), and 4. Fiber intake was significantly below adequate levels in all meal plans (<1%). Unsurprisingly, dairy inclusion resulted in higher calcium intake in both male and female meal plans, but it was still slightly below the RDI of 1000 mg/day. The inclusion of liver exceeded the RDI for iron intake in the female plan, and the inclusion of iodized salt exceeded the RDI for iodine. In summary, the carnivore diet variations in this study came close to being nutritionally complete when fish and dairy were included. The uniform lack of fiber raises questions on how plant avoiders might fare compared with plant consumers in terms of long-term cardiometabolic and gut health [125–129].

*In summary, although there is an emerging body of observational literature on carnivore diets, there is a scarcity of evidence, besides anecdotal, to support it as a universal solution to diet-related public health issues. It is not clear if the potential benefits are due to the diet itself (i.e., what is consumed) versus what is not consumed (i.e., no “junk” food is consumed; food that is calorically dense, but nutrient-sparse). Furthermore, it is noteworthy that meal plans may fall below the recommended dietary intakes for certain vitamins and minerals.*

### 1.11. Is there a diet that is best for healthy aging and longevity?

Diet represents a potent modifiable factor influencing not only lifespan but also healthspan. Healthspan can be defined as the years lived in good health, free from significant chronic diseases and disabilities, while retaining independence and physical, cognitive, and emotional health. The quality of one's diet significantly impacts biological aging processes through various mechanisms, including modulation of inflammation and oxidative stress [130,131]. Epidemiological studies and clinical trials consistently demonstrate that dietary choices are critical in promoting longevity and maintaining quality of life during aging [130–132].

Specific dietary patterns have emerged as particularly beneficial. The Mediterranean diet, characterized by high intakes of fruits, vegetables, whole grains, legumes, nuts, fish, and olive oil, with relatively low consumption of ultra-processed foods and processed meats, is strongly associated with reduced all-cause mortality and cardiovascular events [131,133,134]. The PREDIMED trial showed that a Mediterranean diet supplemented with extra-virgin olive oil or nuts significantly reduced the incidence of major cardiovascular events in individuals at high risk [133]. Data further support an inverse relationship between adherence to the Mediterranean diet and all-cause mortality [134].

Similarly, the traditional Okinawan diet, historically low in calories but rich in nutrient-dense foods such as sweet potatoes and soy, has been linked to longevity and reduced morbidity observed in the Okinawan population [135]. Evidence suggests this population experienced long-term caloric restriction (CR) without malnutrition, potentially contributing to their healthy aging phenotype [135].

Beyond specific patterns, overall diet quality, emphasizing nutrient-dense foods and adequate protein intake, is crucial [130,135–137]. Sufficient high-quality protein (~1.0–1.5 g/kg/day for older adults, with no evidence showing that more is harmful) plays a vital role in preserving skeletal muscle mass and function, thereby maintaining strength and independence, which are key components of healthspan [136,137].

The overall quality and nutritional adequacy of the diet play pivotal roles in determining both lifespan and healthspan [130,131]. Diets characterized by high intakes of nutrient-dense foods such as fruits, vegetables, nuts, fish, etc., are consistently associated with lower rates of all-cause mortality and a reduced incidence of major age-related diseases, including cardiovascular disease (CVD) and certain cancers [130,131,138,139]. Conversely, dietary patterns high in processed meats, refined carbohydrates, added sugars, and excess saturated fats have been linked to increased chronic disease risk and potentially accelerated aging [130].

Compelling evidence from large prospective cohort studies underscores this relationship [130,134,138]. High adherence to quality diets demonstrates inverse associations specifically with CVD mortality and, in some populations or specific patterns, with cancer mortality as well [131,139]. These findings highlight that the benefits for longevity and healthspan are strongly tied to the overall quality and composition of the diet, rather than strict adherence to specific fad diets [130,134,138].

In older adults, intentional weight loss combined with exercise improves physical function [140]. Excess body weight, particularly increased abdominal adiposity, is recognized as exacerbating the age-related decline in physical function and can promote conditions associated with accelerated aging, such as chronic inflammation and metabolic dysfunction [130,140].

Conversely, moderate CR without malnutrition has been demonstrated to extend lifespan and healthspan across various species [135]. Epidemiological evidence from populations like traditional Okinawans, who historically consumed a low-calorie, nutrient-dense diet, suggests a link between their lifelong lower body mass index (BMI), potential CR phenotype, and longevity [135]. Interventions combining intentional weight loss (around 10%) with exercise in obese older adults have proven effective in improving physical function, further highlighting the importance of managing weight in aging populations [140]. These findings underscore the significance of achieving and maintaining a healthy weight, often through calorie moderation paired with nutrient-dense food choices and exercise, for promoting healthy aging [130,135,140].

Preserving skeletal muscle mass and physical function is fundamental for maintaining independence and optimizing quality of life during aging [136,137]. The age-related loss of muscle mass and strength, known as sarcopenia, significantly contributes to disability, falls, reduced mobility, and increased mortality risk [136]. While multiple factors contribute to sarcopenia, such as a sedentary lifestyle, inadequate dietary protein intake is recognized as a key factor that can accelerate this process [136,137].

Current evidence strongly suggests that protein intake above the standard recommended dietary allowance (RDA) of 0.8 g/kg/day is beneficial for older adults [136,137]. Research, including recommendations from the PROT-AGE Study Group, indicates optimal intakes are likely between 1.0–1.5 g/kg/day to effectively preserve muscle mass and function, depending on health status [136]. Consuming sufficient high-quality protein, rich in essential amino acids like leucine, appears critical for stimulating muscle protein synthesis [136,137]. Studies suggest that a per-meal threshold of approximately 25–30 g of high-quality protein may be helpful in maximizing this anabolic response in older individuals [136]. Higher protein consumption (around 1.1–1.2 g/kg/day) has been associated with significantly less lean mass loss over time compared to lower intakes (around 0.7–0.8 g/kg/day) in community-dwelling older adults [141].

Furthermore, combining higher dietary protein intake with regular exercise, particularly resistance training, maximizes muscle preservation and functional outcomes [136,140]. Exercise enhances the muscle's sensitivity to the anabolic effects of amino acids, making protein consumption around the time of physical activity potentially more effective [136]. Therefore, adequate protein intake, especially from high-quality sources, alongside regular physical activity, is crucial for mitigating sarcopenia and extending healthspan [136,137].

Fundamental strategies supported by scientific evidence include:

- Emphasis on diet quality: to support lower rates of all-cause mortality and age-related diseases, prioritize high intake of nutrient-dense, minimally processed foods rich in antioxidants, fiber, and unsaturated fats, such as fruits, vegetables, whole grains, legumes, nuts, and fish [130,131,134,135,138,139,142].
- Caloric moderation: strategies like sustained mild caloric restriction (without malnutrition), as observed historically in long-lived populations, appear beneficial for metabolic health and potentially slowing aging processes [130,135].
- Adequate protein intake: ensure sufficient consumption of high-quality protein, potentially exceeding the standard RDA for older adults (aiming for a minimum of 1.0–1.5 g/kg/day), to preserve muscle mass and function, thus combating sarcopenia and supporting independence [136,137]. Lifting weights and distributing protein intake across meals may optimize muscle protein synthesis [136,137].
- Limiting excess potentially detrimental foods: avoid or limit the overconsumption of foods associated with hyperpalatability and metabolic dysfunction, particularly ultra-processed products characterized by foods high in added sugars, refined grains, and excess saturated and trans fats [130,131,138,142]. A higher intake of processed meats and an insufficient intake of plant foods, for example, has been associated with an increased mortality risk [138].

Ultimately, focusing on these foundational principles, such as emphasizing whole, nutrient-rich foods, ensuring protein adequacy, and practicing caloric moderation, is a reasonable strategy for a long and healthy life [131,135,136].

It is worth noting that nutritional epidemiology may be prone to substantive errors. The primary issue is the reliance on self-reported dietary data. This is often inaccurate due to underreporting, social desirability bias, and poor recall [143]. Thus, it is important to keep that in mind when relying on observational data. On the other hand, randomized controlled trials on various diets suffer from insufficient duration. Does studying the effects of a dietary pattern for several weeks or months reflect what occurs over the course of a lifetime? Overall, one must interpret observational studies with considerable caution.

*In summary, current evidence supports the benefits of high-quality diets, caloric moderation, and adequate protein intake for healthy aging. Future studies should explore the optimal quantity, quality, and timing of protein intake across different life stages and health conditions, ideally examining outcomes beyond traditional biomarkers. Beyond diet, social factors such as community and support also play a significant role in longevity.*

### 1.12. Is there such a thing as “Superfoods”?

The term superfood is defined as a nutrient-dense food considered to have beneficial effects on health and “well-being” [144]. Superfoods are often recognized for high concentrations of single nutrients, vitamins, minerals, and antioxidants beyond basic nutritional needs. Superfoods initially emerged as part of an advertising and marketing campaign [145]. Thus, the term superfood is a marketing term and is often used in place of “functional food.” Additionally, superfoods, or more precisely, superfruits, typically characterize exotic fruits that are touted to have high concentrations of anthocyanins, flavonoids, phenolics, dietary fiber, and often omega-3 fatty acids [146]. In contrast, nutrient-dense foods are foods that provide a high concentration of vitamins, minerals, and micronutrients relative to their caloric load; nutrient-dense foods, when consumed consistently in a well-rounded diet, have been shown to contribute to overall wellness, overall energy, and disease prevention [147,148]. Dietary patterns and consistent macro and micronutrient intake impact overall health and well-being—beyond a single “superfood.”

Beneficial nutrient-dense foods commonly listed as superfoods that may enhance exercise performance and recovery include beets, tart cherries, blueberries, chia seeds, and spinach, to name a few. For instance, beetroot is rich in dietary nitrates, which have been shown to improve oxygen efficiency and endurance during exercise by enhancing nitric oxide production [149–151]. Studies highlight its role in reducing oxygen cost during submaximal exercise and improving time-to-exhaustion [149]. Similarly, tart cherries, abundant in anthocyanins, exhibit anti-inflammatory and antioxidant properties, aiding in muscle recovery and reducing exercise-induced muscle soreness [152–154]. Omega-3 fatty acids, found in salmon and chia seeds, support cellular integrity and anti-inflammation, which are important aspects of recovery [155,156]. Additionally, foods like quinoa and spinach provide essential micronutrients such as magnesium and iron, which are critical for energy metabolism and oxygen transport [157,158], respectively, but by themselves are not “super.” Blueberries are often touted for their antioxidant and polyphenol content, which may help mitigate oxidative stress from exercise and potentially promote faster recovery; however, minimal data on these effects exist, and current evidence demonstrates no significant impact on exercise performance or recovery in humans [159]. Conversely, McLeay et al. found that consuming a blueberry smoothie before and after exercise-induced muscle damage enhances the recovery of peak isometric strength [160]. It is important to note that many of the documented benefits from these foods are often from concentrated extracts, such as beetroot extract [161] and tart cherry extract; however, the results from consuming these extracts do not result in the same effect as eating the whole “superfood.” Thus, superfoods, while containing beneficial macro- and micronutrients, often cannot have a measurable effect when just consumed through food. Additionally, it is a myth to consider a single food to have significantly superior effects on human health and performance. In contrast, nutrient-dense foods should be consumed as part of a comprehensive nutrition strategy tailored to individual needs, emphasizing variety and balance rather than relying on any single superfood.

*In summary, the term superfood is often used to describe nutrient-dense foods, particularly fruits, vegetables, and seeds. Nonetheless, there is no standard scientific definition of the term itself. The concept likely stems from the fact that consumers have a wide variety of food choices. These include junk food (i.e., nutrient-sparse, calorically dense foods) and superfoods (i.e., nutrient-dense foods). One’s health is influenced by a wide variety of factors, such as physical activity, sleep hygiene, body composition, and diet. Hence, the focus on any single food seems a bit misguided.*

### 1.13. Can one consume adequate protein on a vegan diet?

Protein is an essential macronutrient for a variety of physiological processes [162] and the consumption of adequate dietary protein is important for the maintenance of optimal health [163]. It is well known that animal-based foods are rich in protein, especially the essential amino acid leucine [164]; however, vegan diets, specifically where animal-based and by-product foods are excluded from the diet [64,165,166] have been subject to concerns about the ability to consume adequate amounts of total daily protein and/or high-quality protein (i.e. higher concentrations of essential amino acids).

The main concern with vegan diets is that they are commonly low in overall protein intake [64] compared to other dietary patterns. According to the World Health Organization (WHO), a daily protein intake of 0.83 g/kg is expected to meet the needs of the majority (97.5%) of a healthy adult population [167]; however, special populations such as the elderly or physically active individuals may need a higher intake for optimal performance and recovery. In reviews looking at dietary analyzes of vegans compared to non-vegans, overall protein intake is found to be lower in vegans. In a review by Mariotti & Gardner [164], using data from the EPIC-Oxford study, protein intake in vegans was found to be lower (0.99 g/kg/day) when compared to other diets, including meats (1.28 g/kg/day), seafood (1.17 g/kg/day), and dairy (1.04 g/kg/day). Studies have shown that the protein range for vegans is typically between 50 g to 82 g/day or 11.1% and 14.1% of overall energy intake [164]. However, to compensate for this protein deficit, vegans can and often consume higher amounts of plant-based protein foods (i.e. tofu, soy) [168] to increase overall protein intake. For example, in a cohort study by Papier et al. [166], it was found that low and non-meat-eaters consumed higher amounts of high-protein meat alternatives (i.e. soy, legumes, pulses, nuts, and seeds) compared to higher animal-protein diets.

In addition to protein quantity, amino acid composition and the bioavailability of protein sources are also concerns, as there are notable differences in the digestibility of plant-based proteins. Protein quality is determined by the digestibility of the amino acid content [165], and some plant-based protein sources (i.e. soy protein, gluten) have a high digestibility rating (>95%), which is similar to animal-based protein sources [165]; however, other plant-based protein sources are substantially lower (50%–80%). This factor should be considered when determining overall protein needs, especially in populations with higher protein requirements. The amino acid profile, specifically the distribution of essential amino acids, in plant-based foods may be less optimal than in animal-based foods; however, the amounts and proportions of amino acids are sufficient when a variety of food choices are consumed [164].

Overall, the evidence supports the conclusion that one can consume adequate protein on a vegan diet. Special attention should be made to ensure an adequate amount of high-quality plant-based protein sources are consumed from a variety of different sources to ensure individuals consume the required protein intake amount for the individual, as well as sufficient intake of other specific nutrients (iron, zinc, vitamin B12, etc.), which may also be a concern [64,168] when following a plant-based diet.

*In summary, a well-planned vegan diet with a focus on consuming a variety of high-protein plant foods can comply with nutritional recommendations, including protein intake* [166]. *“It should be noted that one can compensate for the generally lower digestibility and suboptimal essential amino acid profiles of many plant-based proteins by consuming a larger total quantity of a variety of complementary plant protein sources (e.g., legumes + grains, soy, pea + rice blends) and/or using fortified or blended plant protein supplements (e.g., soy, pea, and rice protein). This strategy helps meet both total protein and leucine thresholds needed for optimal muscle protein synthesis.*

### 1.14. Do high-protein diets harm the kidneys?

One of the most common and frequently encountered myths or misconceptions is that a high-protein diet contributes to renal and kidney damage in healthy individuals [169]. This misconception persists despite a lack of and often contradictory evidence [6,170,171]. Several studies have demonstrated that increased protein intake does not impair kidney function in individuals without pre-existing kidney disease. For example, a randomized controlled trial by Antonio et al. (2016) examined resistance-trained males ingesting a high-protein diet ranging between ~2.51 and 3.32 g/kg/day of protein over a one-year period and found no adverse effects on kidney function markers, including blood urea nitrogen (BUN), creatinine, or glomerular filtration rate (GFR) [170]. In addition, Antonio and Ellerbroek conducted a series of case studies on 5 resistance-trained males who consumed high-protein diets (>2.2 g/kg/day) over a 2-year period [172]. A comprehensive metabolic panel was completed, revealing no detrimental effects on liver function (aspartate transaminase and alanine transaminase) or kidney function (BUN, creatinine, and eGFR). Similarly, a systematic review by Devries et al. (2018) concluded that high-protein diets (≥1.5 g/kg/day or ≥20% energy intake from protein or ≥100 g of protein/day) compared to normal or lower protein intakes do not negatively affect kidney health (i.e. change in glomerular filtration rate [SMD: 0.11; 95% CI: −0.05, 0.27, *p* = 0.16]) in healthy adults without kidney disease [171]. This systematic review included 28 randomized controlled trials with 1358 participants and provides robust evidence that a higher protein diet in healthy individuals does not cause kidney damage.

Strength-based athletes often consume higher amounts of dietary protein, and currently, there is no evidence of an increased risk in this population [6,80,173]. For example, Poortmans and Dellalieux (2000) demonstrated that high protein intakes did not impair renal function in bodybuilders or other well-trained athletes [174]. They found higher concentrations of uric acid and calcium; however, the clearances of creatinine, urea, and albumin were all within normal ranges. The authors concluded that protein intakes under 2.8 g/kg/day did not impair renal function in well-trained athletes. In a recent review of nutritional strategies used by bodybuilders on kidney function, the authors concluded that there is little evidence that high-protein diets pose any risk to individuals with normal kidney function [173].

Multiple meta-analyzes and RCTs show that high-protein diets increase GFR, a marker of hyperfiltration, but do not cause adverse changes in serum creatinine, albuminuria, or other markers of kidney damage over periods ranging from weeks to two years [175–181]. These changes are considered physiological adaptations rather than pathological injury [182,183].

Additionally, the Institute of Medicine and the World Health Organization report on protein states that there is no evidence linking higher protein diets to renal disease [167,184]. Moreover, a panel charged with establishing reference intakes for Australia and New Zealand [185] stated that there is no scientifically published evidence demonstrating that a diet containing up to 2.8 g/kg/day of protein causes adverse renal effects in healthy adults.

*In summary, current research strongly supports the conclusion that higher-protein diets do not impair kidney function in healthy humans over short- to medium-term durations; observed increases in GFR are adaptive rather than harmful.*

### 1.15. Does a greater meal frequency lead to greater weight loss?

It has been widely believed that consuming smaller, more frequent meals throughout the day promotes greater weight loss compared to eating fewer, larger meals. “Ramping up your metabolic furnace” or “stoking the flames of weight loss” are all sayings that have been shared in reference to the rationale or justification why an increased meal frequency may promote greater weight loss and body composition changes. Early observational research offering preliminary support for this recommendation comes from Hejda and Fabry [186] who observed that in a cohort of 379 overweight adults aged 60–64 years, there was an inverse relationship between meal frequency and average skinfold thickness—a surrogate measure of body fat. This finding suggests that individuals who consume meals more frequently tend to have lower body fat levels. Similarly, a subsequent study by Metzner et al. [187]. In a larger population sample of 2000 adults aged 35–60 years reported an inverse association between meal frequency and adiposity was reported.

Despite their intriguing findings, these observational studies were limited by methodological weaknesses that likely influenced the outcomes. More rigorous investigations using randomized controlled trial designs have since challenged these earlier conclusions. For instance, Cameron et al. [188] assigned 16 obese participants to consume either two or six meals per day while reducing their daily caloric intake by 700 kcal over an eight-week period. Results showed no significant differences between groups in weight loss, body fat changes, or biomarkers relevant to weight-loss outcomes. Similarly, in a two-week crossover study by Kulovitz et al. [189], participants followed a calorie-restricted diet while alternating between consuming their daily intake as either two or six meals. Once again, no meaningful differences in weight loss, body composition, or metabolic biomarkers were found. These and other comprehensive reviews [190,191] have reinforced the notion that it is not the number of meals or the energy or macronutrient load of those meals, but rather the total energy deficit achieved and maintained, that most strongly impacts changes in body weight and composition.

While meal frequency may not significantly affect weight-loss potential, it is worth noting that more frequent meals can offer benefits beyond weight loss. For example, numerous studies have reported improvements in cardiometabolic risk markers such as total and LDL cholesterol and insulin sensitivity, with higher meal frequencies. Furthermore, increased meal frequency appears to aid in hunger and appetite regulation and may help people to achieve a more consistent energy deficit. Finally, greater meal frequency and smaller meal size are often associated with reduced gastrointestinal discomfort [191]. These results provide some health-related reasons why someone may choose to consume smaller, more frequent meals.

*In summary, while meal frequency may influence certain health-related parameters, especially metabolic and subjective outcomes, the primary determinant of weight loss and improvements in body composition remains the successful creation and maintenance of an energy deficit.*

### 1.16. Are carbohydrates more fattening than dietary fats?

Both carbohydrates and fats play essential roles in human energy metabolism [81,192]. The belief that carbohydrates are inherently more fattening than dietary fats stems from longstanding misconceptions about insulin, simplified views of calorie storage, and the popularity of low-carbohydrate diet trends. This section aims to evaluate the scientific evidence on whether carbohydrates are, in fact, inherently more fattening than dietary fats. For this section, the term “fattening” refers to the promotion of fat mass through the storage of excess calories.

Carbohydrates are one of the three primary macronutrients, alongside fats and proteins, that provide energy for the human body. Chemically, they are composed of carbon, hydrogen, and oxygen, and are classified based on their structure into simple carbohydrates, complex carbohydrates, and fiber [193]. Carbohydrates provide four kilocalories per gram. Dietary fats, another essential macronutrient, also supply energy and support a range of vital physiological functions [194]. The majority of dietary fats are in the form of triglycerides, molecules composed of three fatty acids attached to a glycerol backbone [195]. These compounds are a dense source of energy, supplying nine kilocalories per gram, more than twice the energy provided by carbohydrates or proteins. Triglycerides are further classified based on the length of their fatty acid chains and their degree of saturation (i.e. the presence or absence of double bonds) into saturated and unsaturated fats.

In the 1980s, public health guidelines in the United States promoted low-fat diets as a strategy to reduce heart disease and improve overall health [196]. As a result, the food industry reformulated many products to be low in fat but often replaced the fat with refined carbohydrates and added sugars to maintain taste and texture. This shift contributed to the widespread consumption of highly processed, calorie-dense foods, which may have inadvertently fueled the rise in obesity and metabolic disorders despite the reduction in total fat intake. By the late 1990s and early 2000s, dissatisfaction with the effectiveness of low-fat diets led to the resurgence of low-carbohydrate diets, such as Atkins, South Beach, and, more recently, ketogenic diets [197,198]. These eating patterns prioritize fat and protein while restricting carbohydrate intake, claiming superior benefits for weight loss and metabolic health. As these diets gained popularity, the public perception of macronutrients shifted: carbohydrates became widely demonized as the primary drivers of weight gain and insulin resistance, while fats, once vilified, became partially rehabilitated. This pendulum swing in dietary beliefs set the stage for ongoing confusion and debate over which macronutrient is more inherently fattening, a question that requires careful evaluation of scientific evidence and consideration of broader dietary context.

The principle of energy balance, the relationship between calories consumed (“calories in”) and calories expended (“calories out”), is fundamental to understanding weight gain and loss. While all calories contribute to energy intake, they are not metabolized in the same manner. For instance, the thermic effect of food, the energy required to digest, absorb, and process nutrients, is slightly higher for carbohydrates than for fats, meaning fewer net calories are stored from carbohydrates [199]. Additionally, carbohydrates and fats differ in their effects on satiety and hormones [200,201]. Carbohydrates, particularly those high in fiber, can promote feelings of fullness while also stimulating the release of insulin, a hormone involved in nutrient storage. While insulin does promote fat storage in the presence of excess energy, it has been mischaracterized as the sole cause of weight gain. A tightly controlled inpatient metabolic ward study by Hall et al. [202] found that calorie for calorie, restricting dietary fat led to greater body fat loss than restricting carbohydrates in adults with obesity. This occurred even though only the low-carbohydrate diet reduced insulin levels and increased fat oxidation. These findings challenge the widely held notion that carbohydrate restriction provides a metabolic advantage for fat loss. Instead, the results suggest that fat loss is primarily driven by caloric deficit, not insulin suppression, and that carbohydrate restriction is not necessary for reducing body fat [202–204].

In addition to controlled metabolic studies, longer-term trials have also investigated the effects of macronutrient composition on weight loss. An updated review of 61 randomized controlled trials involving 6925 men and women compared low-carbohydrate diets (less than 50 g/day) to balanced-carbohydrate diets (up to 65% of total energy intake) over periods ranging from 12 weeks to two years [205]. The review found that low-carbohydrate diets led to little to no difference in body weight change compared to balanced-carbohydrate diets, both in the short term (i.e. months) and long term (i.e. years). These findings were consistent across individuals with and without type 2 diabetes.

*In summary, the evidence does not support the claim that carbohydrates are inherently more fattening than fats. Instead, total calorie intake and overall diet quality remain the primary determinants of weight gain. Weight gain is primarily driven by the overconsumption of energy-dense, ultra-processed foods that are often high in both refined carbohydrates and fats* [206]*. Large portion sizes, low satiety, and an obesogenic food environment further contribute to chronic excess calorie intake* [207]*. Moreover, neither carbohydrate nor fat is inherently fattening in the absence of a caloric surplus.*

## 2. Conclusions

Based on our evidence-based scientific evaluation of the literature, we conclude the following.

1. Hypercaloric strategies can promote skeletal muscle gain. Nonetheless, outcomes vary with training status, surplus size, and macronutrient composition. Notably, beginners and those at an intermediate level can often improve their body composition by simultaneously gaining lean mass and losing fat. In practice, aiming for a 10%–20% calorie surplus, with a focus on protein intake, is an effective way to promote lean mass gain while minimizing fat accumulation.
2. Although creating a caloric deficit remains ideal for optimal weight management, substantial evidence indicates that modifying dietary macronutrient distribution can independently facilitate significant body weight and fat loss.
3. Both intermittent fasting (IF) and chronic caloric restriction (CCR) are effective strategies for weight loss, showing only modest differences in short-term results. Choosing between IF and CCR should be based on individual needs, preferences, and metabolic health.
4. Although some sex differences have been noted in studies on intermittent fasting, with or without exercise, the patterns, strength of the effects, and significance of these differences are not clear enough to definitively say whether fasting programs are “better” or “worse” for women compared to men.
5. Many athletes fail to consistently meet their required intake of macro- and micronutrients. As a result, relying solely on diet is often insufficient for meeting the nutritional demands of most individuals who regularly exercise. It is important to thoughtfully select supplements to address any nutritional gaps. Although prioritizing whole foods is preferable, most athletes find it challenging to meet their heightened nutrient requirements solely through diet.
6. Research increasingly supports a more personalized approach that emphasizes overall calorie balance, sufficient daily protein, nutrient-rich foods, and alignment with individual health and performance goals. Instead of strictly set macronutrient percentages (e.g. 40:30:30), dietary plans should be designed to enhance outcomes such as performance, recovery, and long-term well-being, while allowing flexibility based on each person's unique needs and goals.
7. Research indicates that the primary benefits of most diets for overweight and obese individuals come from weight loss achieved through calorie restriction, rather than the specific makeup of the diet. However, including adequate protein can enhance the positive effects of a weight-loss plan.
8. Research demonstrates that when calories and protein intake are matched, both high-carbohydrate/low-fat and low-carbohydrate/high-fat diets result in similar weight loss, with only minor differences between groups. Moreover, there is currently no evidence that diets tailored to a person's DNA offer any extra advantages over conventional dietary strategies.
9. The preponderance of evidence does not support the common belief that eating late at night inevitably leads to fat gain, and highlights the importance of a more sophisticated approach to nutrient timing for improving metabolic health and body composition. One might posit that the composition of late-night eating is a critical factor regarding potential fat gain (i.e. a high-protein snack versus a high-carbohydrate/fat snack).
10. Observational studies on carnivore diets are increasing; however, there is still little robust scientific evidence to support it as a universal solution for diet-related public health concerns. Some anecdotal accounts report health improvements with the carnivore diet, but it remains unclear whether these benefits come from the foods included or from the exclusion of processed, calorie-dense, nutrient-poor foods. Perhaps it is a combination of both.
11. Existing research highlights the positive impact of nutrient-rich diets, controlled calorie intake, and sufficient protein on healthy aging; however, further investigation is needed. Future studies should focus on determining the optimal amounts, types, and timing of protein for various age groups and health conditions, and should also consider outcomes beyond standard health markers. Perhaps more importantly, factors such as social connections and support play a crucial role in promoting longevity.
12. The term “superfood” is commonly used to describe foods rich in nutrients, particularly fruits, vegetables, and seeds. However, there is no official scientific definition for this label. The idea arises from the vast range of food options available to consumers, from nutrient-poor, high-calorie junk foods to nutrient-dense choices labeled as superfoods. Overall health is shaped by many factors, such as physical activity, sleep quality, body composition, and diet. Placing too much emphasis on any single food is somewhat misplaced.
13. A carefully designed vegan diet that includes a diverse range of protein-rich plant foods can meet nutritional guidelines, including adequate protein intake. It's important to recognize that any shortcomings in the quality of plant-based proteins can be offset by eating greater amounts of these protein sources.
14. There is no evidence that consuming a high-protein diet, whether it is from whole foods or supplements, has an adverse effect on kidney function in otherwise healthy individuals.
15. Meal frequency can affect some health factors, particularly metabolic and personal perceptions. The key factor for achieving weight loss and improving body composition is consistently creating and maintaining an energy deficit.
16. Current evidence does not back the notion that carbohydrates are naturally more likely to cause weight gain than dietary fats under conditions in which total calories and protein intake are equivalent. Rather, it is the total number of calories consumed and the overall nutritional quality of the diet that most strongly influence weight gain.
